# Static Magnetic Field Accelerates Diabetic Wound Healing by Facilitating Resolution of Inflammation

**DOI:** 10.1155/2019/5641271

**Published:** 2019-11-30

**Authors:** Wenlong Shang, Guilin Chen, Yinxiu Li, Yujuan Zhuo, Yuhong Wang, Zhicai Fang, Ying Yu, Huiwen Ren

**Affiliations:** ^1^Department of Pharmacology, Key Laboratory of Immune Microenvironment and Disease (Ministry of Education), School of Basic Medical Sciences, Tianjin Medical University, Tianjin, China; ^2^Heye Health Industrial Research Institute of Zhejiang Heye Health Technology, Anji, Zhejiang 313300, China

## Abstract

Impaired wound healing is commonly encountered in patients with diabetes mellitus, which may lead to severe outcomes such as amputation, if untreated timely. Macrophage plays a critical role in the healing process including the resolution phase. Although magnetic therapy is known to improve microcirculation, its effect on wound healing remains uncertain. In the present study, we found that 0.6 T static magnetic field (SMF) significantly accelerated wound closure and elevated reepithelialization and revascularization in diabetic mice. Notably, SMF promoted the wound healing by skewing the macrophage polarization towards M2 phenotype, thus facilitating the resolution of inflammation. In addition, SMF upregulated anti-inflammatory gene expression via activating STAT6 and suppressing STAT1 in macrophage. Taken together, our results indicate that SMF may be a promising adjuvant therapeutic tool for treating diabetic wounds.

## 1. Introduction

The diabetic foot ulcer is one of the most common and severe complications of diabetes mellitus because of impaired wound healing [1, 2]. More than one million diabetes patients have to undergo lower limb amputation per year worldwide [3], which makes up approximately 50%–70% of all limb amputations. The standard treatment for diabetic wounds includes debridement of the wound, treatment of any infection, revascularization, and off-loading of the ulcer [4]. Although several strategies, such as the wound healing peptides, have been used with high efficiency [5, 6], some refractory wounds and high costs of wound care predispose the patients to delay the treatment. Thus, it is desirable to explore alternative and cost-effective therapies for the patients with severe diabetic wounds.

Static magnetic field (SMF) has been applied in medicine as a tool to increase bone regeneration and promote drug delivery [7, 8]. Accumulating evidences have demonstrated multiple beneficial effects of magnetic therapy, such as the recovery of the soft tissue and nerve system injury and insomnia [9–13]. Studies have also shown that SMF may influence the production of inflammatory cytokines released by macrophages and lymphocytes [14]. However, the therapeutic effect of SMF on diabetic wound healing remains to be determined.

During the process of wound healing, macrophages plays a critical role in modulating the inflammation and angiogenesis [15]. Basically, the macrophages are classified into two phenotypes: the “classically” activated macrophage (M1) and “alternatively” activated macrophage (M2) [16]. The M1 macrophage exhibits a proinflammatory function and promotes bacterial clearance and host defense by increasing phagocytosis and the production of inflammatory cytokines, while the M2 macrophage facilitates the resolution of inflammation and angiogenesis and promotes tissue remodeling by releasing anti-inflammatory cytokines and growth cytokines [17–19]. *In vitro*, SMF suppresses the production of inflammatory cytokines released by macrophages and lymphocytes [14]. However, the mechanism underlying SMF-mediated regulation of inflammation reaction is still unclear.

In this study, we observed that SMF significantly accelerated wound closure and revascularization by driving macrophages towards M2 polarization and inflammatory resolution through balancing STAT1/STAT6 signaling. The results suggest that SMF may serve as an effective therapeutic approach for diabetic wound.

## 2. Methods

### 2.1. Animals

Male BKS-Lepr^em2Cd479^/Nju (db/db) mice at 8-12-week old were used in this study. All mice were purchased from GemPharmatech Co. Ltd. and were maintained in mouse barrier facilities of Tianjin Medical University. All *in vivo* experiments complied with the Guidelines of the Institutional Animal Care and Use Committee of Tianjin Medical University that approved all protocols.

### 2.2. Wound Healing Model

Mice were anesthetized by inhalation of isoflurane; the dorsal surface was shaved, washed with povidone iodine solution, and cleaned with an alcohol swab. Two excisional wounds were made on each side of the midline of the shaved dorsum using a sterile 8-mm punch biopsy tool (Miltex, USA). The wounds were covered with self-adhesive dressings (Cofoe). Diabetic mice with excisional wounds were housed on the top of the magnetic or nonmagnetic plate (230 mm × 130 mm × 15 mm) within the cage. Wound sizes were monitored under Leica Microsystems (Leica Microsystems Ltd.) and calculated using ImageJ software (National Institutes of Health). Injured skin tissues were subjected to paraffin embedding, serial sectioning, and subsequent hematoxylin and eosin (H&E) staining. Then, wound healing was assessed by measuring the largest distances between epithelial tips or panniculus carnosus edges in H&E-stained tissue using CaseViewer (3DHISTECH) [20].

### 2.3. Immunofluorescence Staining

For immunofluorescence staining, deparaffinized and dehydrated sections (5 *μ*m) of the wounds from db/db mice were fixed in 4% paraformaldehyde. The slides were treated with 0.25% Triton X-100 in PBS for 30 min for permeabilization and blocked with 5% goat serum for 1 h. The sections were then incubated with primary antibodies: Mac-3 (diluted 1 : 200, BioLegend, 108512), iNOS (diluted 1 : 50, Abcam, ab15323), Arginase-1 (Arg-1, diluted 1 : 200, Proteintech, 16001-1-AP), or CD31 (diluted 1 : 50, Abcam, ab28364) at 4°C overnight. The samples were washed with PBS and incubated with secondary antibodies: Alexa Fluor 488 goat anti-rabbit IgG (1 : 1000, Invitrogen) and Alex Fluor 633 goat anti-mouse IgG (1 : 1000, Invitrogen) for 2 h at room temperature. The sections were then counterstained with DAPI and sealed with the antifade reagent. Immunofluorescence images were captured using a Zeiss laser scanning confocal microscope at 200x. All images were analyzed with the Image-Pro Plus software (v.6).

### 2.4. Peritoneal Macrophage Isolation and Treatment

The peritoneal macrophages were induced by intraperitoneal injection of 3% Brewer's thioglycolate as described previously [21]. The macrophages were allowed to adhere at 37°C overnight under 5% CO_2_, and unattached cells were removed by washing with fresh medium before use. Macrophage polarization was induced with 1 *μ*g/ml LPS (Sigma, L2880) or 20 ng/ml IL-4 (Proteintech, 214-14).

### 2.5. Quantitative Real-Time PCR

Total RNA from peritoneal macrophage and skin tissues were extracted using the TRIzol reagent (Invitrogen), and the cDNAs were synthesized using the Reverse Transcription Reagent Kit (Takara Bio Inc.) according to the manufacturer's instruction. The resulting cDNAs were amplified with 40 cycles by real-time PCR. Each sample was analyzed three times and normalized to a reference RNA using *β*-actin as the internal control. Sequences of primers used for real-time PCR to analyze the mouse samples are summarized in Table 1.

### 2.6. Western Blotting

Protein quantification was carried out using the BCA Protein Assay Kit (Pierce). Equal quantities of the proteins were denatured and resolved by 10% SDS-PAGE gels, transferred to nitrocellulose membranes, incubated with 5% skimmed milk for 1 h, and then incubated with the primary antibodies at 4°C overnight. The primary antibodies were diluted as follows: iNOS (1 : 500, Abcam, ab15323), Arg-1 (1 : 5000, Proteintech, 16001-1-AP), phospho-STAT6 (pY641) (1 : 1000, BD, 558241), STAT6 (1 : 1000, ABclonal, A0755), phospho-STAT1 (1 : 1000, Cell Signaling Technology, 8826S), STAT1 (1 : 1000, ABclonal, A12075), and alpha tubulin (1 : 2000, Cell Signaling Technology). The membranes were incubated with the HRP-labeled secondary antibody in blocking buffer for 2 h at room temperature. Blots were developed using an enhanced chemiluminescence reagent (Thermo Fisher Scientific). The relative protein density was quantified using Image J1.44.

### 2.7. Flow Cytometric Analysis

The wound cutaneous samples were collected by 8 mm biopsy punch, minced to 2 mm^2^ section on ice, and digested with dispase II (Roche, 04942078001) and Collagenase (Sigma, C0130) for 2 h at 37°C. The cells were incubated with 1% BSA in PBS containing primary antibodies for 0.5 h at 4°C. The primary antibodies were diluted as follows: PE/cy7-F4/80 (1 : 200; BioLegend, 123114), FITC-CD11b (1 : 200; BioLegend, 101205), and APC-CD206 (1 : 200; BioLegend, 141708). The cells were then washed twice before analysis. Stained cells were processed on a FACSAria flow cytometer (BD). The final data were analyzed using FlowJo (v.9; Tree Star).

### 2.8. Migration Assay

Macrophage migration was evaluated using a Transwell system (Corning, 3422, NY, USA). Approximately 5 × 10^4^ macrophages were suspended in 100 *μ*l serum-free medium and seeded onto the upper chambers. Then, 500 *μ*l RPMI medium 1640 basic (Gibco) with 30% fetal bovine serum was added to the lower chambers. After incubation for 24 h at 37°C under 5% CO_2_, the medium was removed from the upper chamber and the macrophages on the upper side of the chamber were scraped off with a cotton swab. The cells on the lower side of the upper chamber were fixed, stained with 0.1% crystal violet, photographed, and counted under a microscope (magnification 200x).

### 2.9. Statistical Analyses

Data were analyzed using unpaired Student's *t*-test or repeated measures ANOVA, followed by Fisher's least significant difference analysis for multiple comparisons. *P* < 0.05 was considered statistically significant.

## 3. Results

### 3.1. SMF Accelerates Wound Healing in db/db Mice

**Figure 1:**
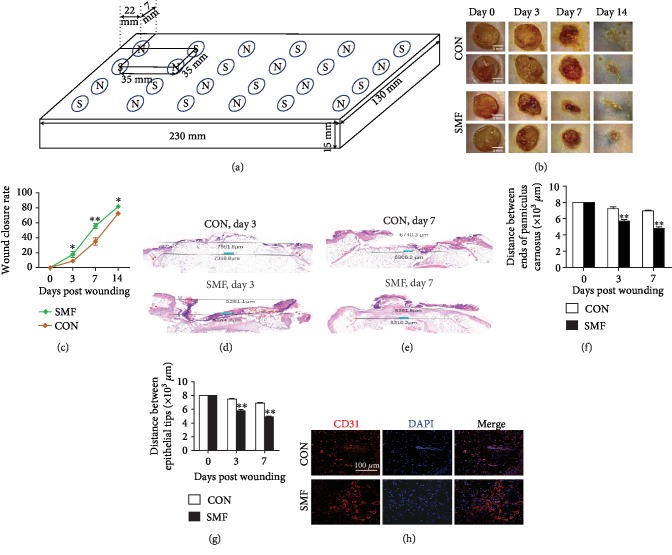
Effect of SMF on diabetic wound healing. (a) Schematic view of the SMF exposure system for diabetic mice. The plate with 24 magnetic pieces (0.6 T) embedded was 230 mm × 130 mm × 15 mm in size. (b) Representative images of excisional wounds of diabetic mice treated with or without SMF. (c) Wound closure rate measurement (*n* = 8 per group). (d, e) Representative H&E staining images of excisional wounds in diabetic mice on days 3 and 7. (f, g) Quantification of the distance between epithelial tips and ends of panniculus carnosus as shown in (d) and (e) (*n* = 4‐8 per group). (h) Representative immunofluorescent staining images of excisional wounds from diabetic mice on day 7. Magnification ×200. Scale bar, 100 *μ*m. Results are expressed as mean ± SEM. (c, f, g) Statistical significance was determined using unpaired Student's *t*-test. ^∗^*P* < 0.05 and ^∗∗^*P* < 0.01 vs. control (CON).

To investigate the therapeutic effect of SMF on diabetic injury, the db/db mice were housed in a 230 mm × 130 mm × 15 mm plate with 24 magnetic pieces (0.6 T) embedded (Figure 1(a)). As shown in Figure 1(b), the SMF treatment promoted wound healing by reducing wound sizes at different time points. Moreover, the wound closure rate in db/db mice exposed to SMF was dramatically higher than that in the control group (Figure 1(c)). Histological analyses revealed significantly shorter distances between the epithelial tips of punched wound and distances between the edges of the panniculus carnosus in the SMF group at day 3 and day 7 postoperatively (Figures 1(d)–1(g)), suggesting that reepithelialization and wound contraction were enhanced in db/db mice exposed to SMF. In addition, the number of CD31-positive cells in the regenerative tissue in SMF-treated mice was notably higher than that in the control group (Figure 1(h)), indicating that SMF enhanced the revascularization in injured tissues.

### 3.2. SMF Promotes Wound Healing by Skewing Macrophage Polarization towards M2 Phenotype

**Figure 2:**
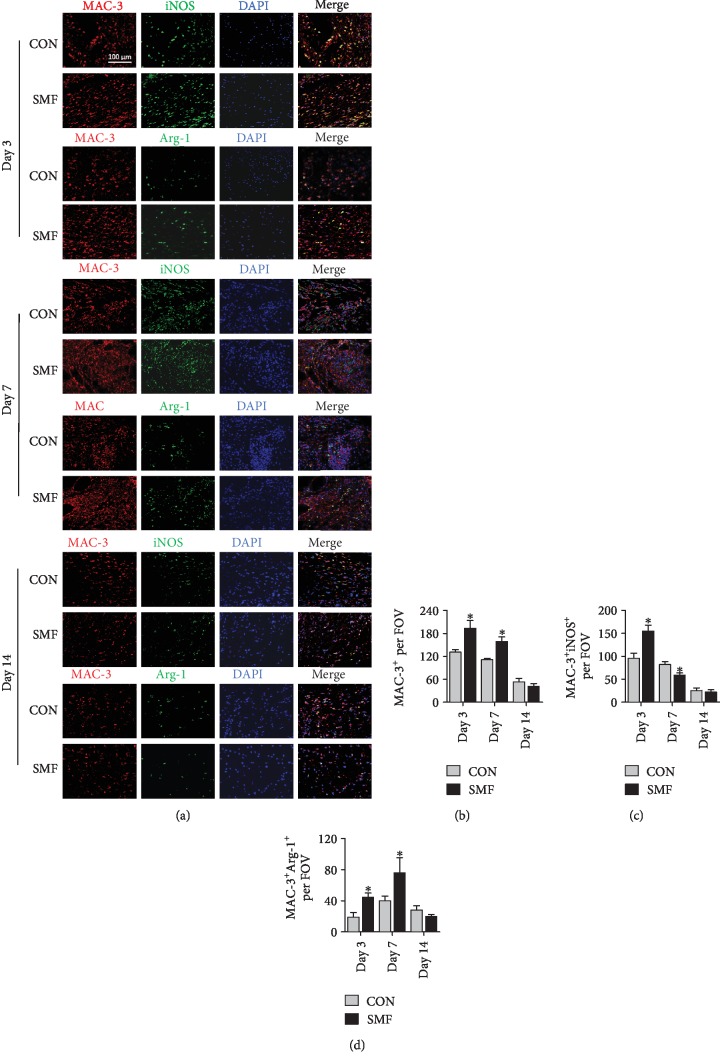
Impact of SMF on macrophage polarization in excisional wounds of diabetic mice. (a) Representative immunofluorescent staining images of excisional wounds in diabetic mice treated with or without SMF on days 3, 7, and 14 after treatment. DAPI (nuclei) = blue, MAC-3 (M0) = red, iNOS (M1) = green, and Arg-1 (M2) = green. Magnification ×200 in macrophage staining. Scale bar 100 *μ*m. (b–d) Quantification of the total macrophages (MAC-3^+^) (b) pro-inflammatory M1(MAC-3^+^/iNOS^+^) (c) and constructive remodeling M2 (MAC-3^+^/Arg-1^+^) (d) macrophages as shown in (a) (*n* = 4‐6 per group). Data are presented as mean ± SEM. (b–d) Statistical significance was determined using unpaired Student's *t*-test. ^∗^*P* < 0.05 and ^∗∗^*P* < 0.01 vs. control (CON).

**Figure 3:**
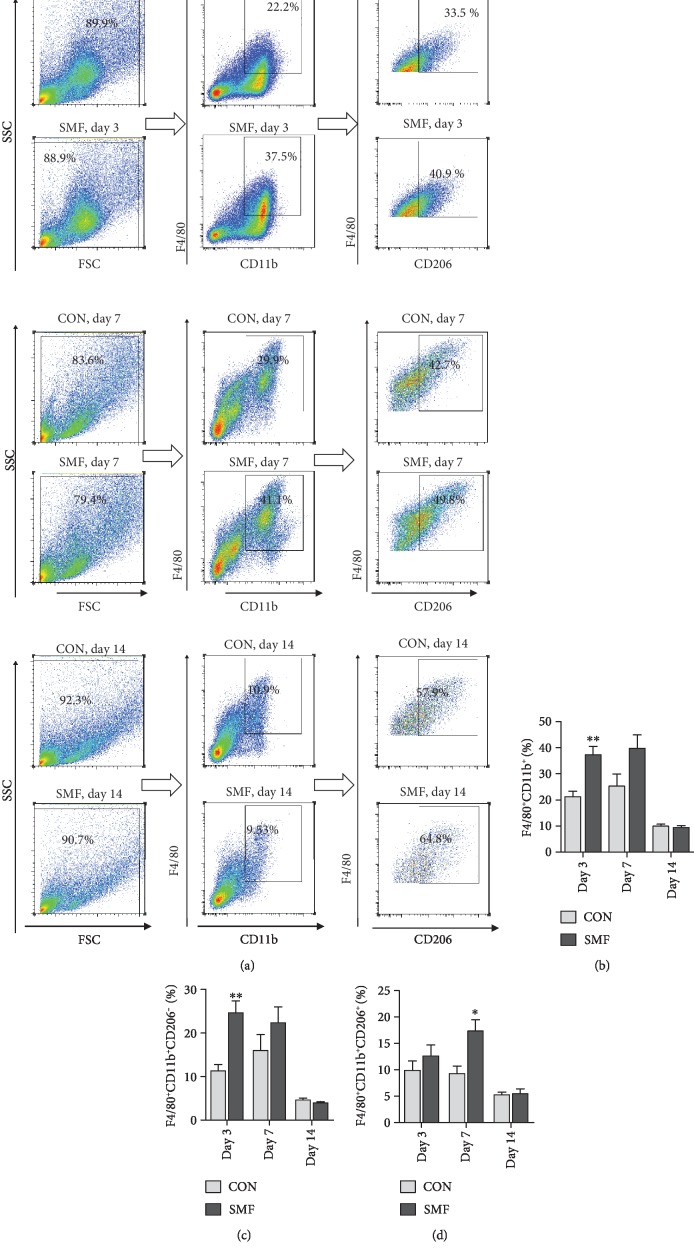
Influence of SMF on the ratio of M1 and M2 macrophage in excisional wounds of diabetic mice. (a) Schematic of flow cytometry gating. (b–d) Quantification of the percentage of total macrophage (F4/80^+^CD11b^+^) (b) M1 macrophage (F4/80^+^CD11b^+^CD206^−^) (c) and M2 macrophage (F4/80^+^CD11b^+^CD206^+^) (d) in cutaneous wounds of diabetic mice treated with or without SMF on days 3, 7, and 14 posttreatment (*n* = 4‐5 per group). Data are shown as mean ± SEM. (b–d) Statistical significance was determined using unpaired Student's *t*-test. ^∗^*P* < 0.05 vs. control (CON).

We first examined the effect of SMF on macrophage infiltration in the injured tissue from db/db mice. SMF treatment significantly increased macrophage recruitment (Mac-3^+^) at day 3 (acute inflammatory phase) and day 7 (tissue regeneration phase) (Figures 2(a) and 2(b)). Interestingly, both M1-like (Mac3^+^iNOS^+^) and M2-like (Mac3^+^Arg-1^+^) macrophages were increased at an early inflammation stage (day 3) by SMF treatment (Figures 2(a)–2(d)). Along with wound healing, SMF promoted M2 macrophage recruitment (Figures 2(a) and 2(d)), while reduced M1 macrophages (Figures 2(a) and 2(c)) were observed in wound areas as seen in db/db mice at day 7, indicating that SMF facilitates M2 polarization in inflamed tissues (Figures 2(a)–2(d)). However, we failed to detect significant difference of M1- or M2-like macrophages at day 14 (at low levels) between SMF-treated and control mice (Figures 2(a)–2(d)). Similarly, flow cytometry analysis revealed that both M1-like macrophages (F4/80^+^CD11b^+^CD206^−^) (Figures 3(a) and 3(c)) and M2-like macrophages (F4/80^+^CD11b^+^CD206^+^) (Figures 3(a) and 3(d)) were increased in the SMF group at day 3 (Figures 3(a)–3(d)). M2-like macrophages in the SMF-treated group were increased significantly compared with those in the control group at day 7 postinjury (Figures 3(a) and 3(d)). Thus, SMF accelerates wound healing in db/db mice by skewing macrophage polarization towards M2 phenotype.

### 3.3. SMF Accelerates Resolution of Inflammation by Promoting M2 Macrophage Polarization in Mice

**Figure 4:**
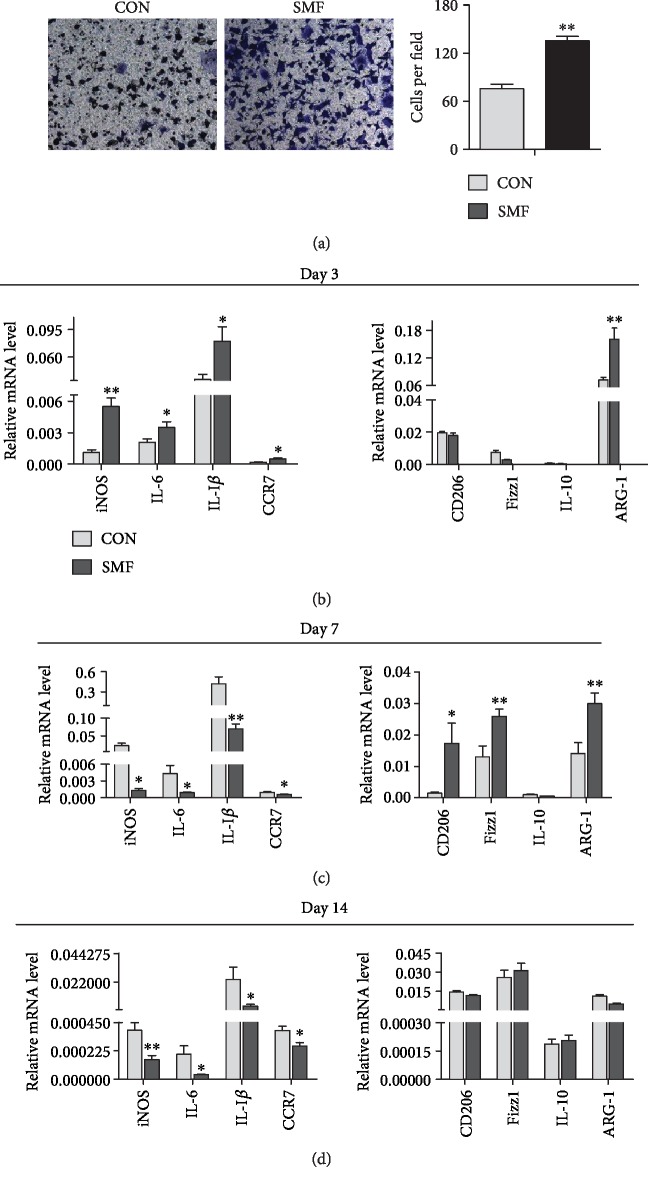
SMF promoted macrophage migration and polarization towards M2 in cutaneous wounds of diabetic mice. (a) The effect of SMF on migration of macrophages (RAW264.7) by transwell migration assay (*n* = 4 per group). Magnification ×200. (b–d) Expression of proinflammatory and anti-inflammatory genes in the cutaneous wounds of diabetic mice treated with or without SMF on days 3, 7, and 14 (*n* = 10‐16 per group). Data are shown as mean ± SEM. (b–d) Statistical significance was determined using unpaired Student's *t*-test. ^∗^*P* < 0.05 and ^∗∗^*P* < 0.01 vs. control (CON).

Consistent with microphage recruitment at an acute inflammation stage (day 3), *in vitro* transwell assay revealed that direct migration of culture macrophages was remarkably enhanced under SMF treatment (Figure 4(a)). We also examined the cytokine expression in wound tissues and found that the expression levels of proinflammatory genes (iNOS, IL-6, IL-I*β*, and CCR7; Figure 4(b)) in injured tissues were elevated at day 3 in SMF-treated mice and paradoxically were downregulated at day 7 and day 14 (Figures 4(c) and 4(d)). Meanwhile, SMF significantly raised the expression levels of reparative genes (CD206, Fizz1, Arg-1, and IL-10) at day 7 (Figure 4(c)). Collectively, these results indicate that SMF promotes inflammation resolution by modulating the expression profile of pro/anti-inflammation cytokines in injured tissues.

### 3.4. SMF Promotes the M2 Macrophage Polarization *In Vitro*

**Figure 5:**
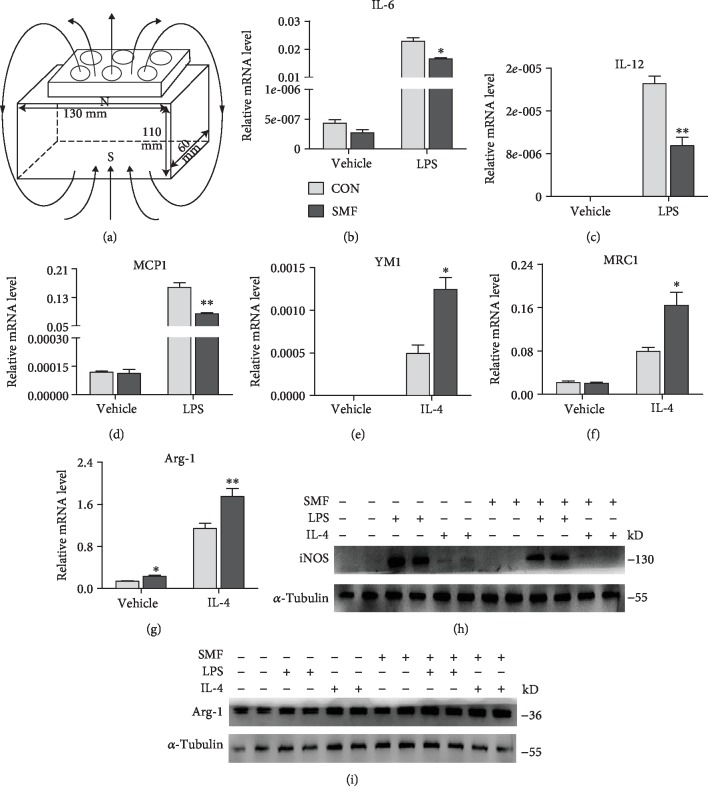
SMF facilitates M2 polarization *in vitro*. (a) Schematic view of the SMF exposure system for cell plates. The size of the plate was 130 × 110 × 60 mm. (b–i) Effect of SMF on macrophage polarization. (b–g) The mRNA level of M1 marker genes (IL-6, IL-12, and MCP1) (b–d) and M2 marker genes (Arg-1, MRC1, and YM1) (e–g) in cultured peritoneal macrophages treated with or without SMF for 24 h (*n* = 3‐4 per group). (h, i) Protein levels of iNOS (M1) (h) and Arg1 (M2) (i) in cultured peritoneal macrophages treated with or without SMF. All graphs are shown as means ± SEM. (b–g) Statistical significance was assayed by unpaired Student's *t*-test. ^∗^*P* < 0.05 and ^∗∗^*P* < 0.01 vs. control (CON).

To examine the impact of SMF on M2 polarization in culture, peritoneal macrophages were isolated from db/db mice and then challenged by LPS or IL-4 to induce M1 or M2 polarization, respectively. After 24 h of treatment with 0.6 T SMF (Figure 5(a)), the RT-PCR assay revealed that proinflammatory cytokines, such as IL-6, IL-12, and MCP1, were markedly downregulated (Figures 5(b)–5(d)). In contrast, anti-inflammatory cytokines, such as YM-1, MRC1, and Arg-1 (Figures 5(e)–5(g)), were significantly upregulated in the SMF group, indicating that SMF suppresses M1 polarization, while promoting M2 polarization. The effects were further confirmed by Western blot assay, which showed a reduction of proinflammatory iNOS expression (Figure 5(h)) and increased expression of anti-inflammatory Arg-1 (Figure 5(i)) in SMF-treated macrophages.

### 3.5. SMF Regulates Macrophage Polarization via STAT1 and STAT6 Activation

**Figure 6:**
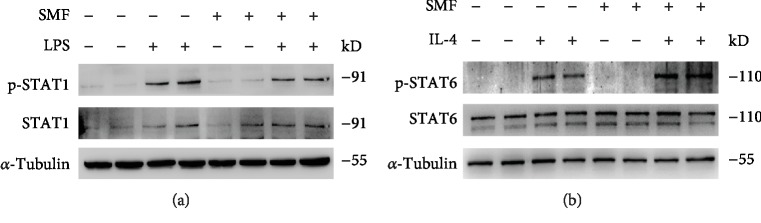
SMF promotes M2 polarization through regulating STAT1/STAT6 activation. (a) The phosphorylation level of STAT1 in LPS-stimulated peritoneal macrophages exposed to SMF for 0.5 h. (b) Expression of p-STAT6 in IL-4-treated peritoneal macrophage after SMF exposure of 0.5 h.

Canonically, LPS/TLR signaling skews macrophage towards the M1 phenotype by activation of STAT1 and IL-4 promotes macrophage towards the M2 phenotype via STAT6. As expected, SMF inhibited LPS-induced STAT1 phosphorylation (Figure 6(a)), while augmented IL-4-induced STAT6 phosphorylation was observed in the macrophages (Figure 6(b)). These data suggested that SMF promotes the macrophage polarization by regulation of STAT1/STAT6 activation.

## 4. Discussion

Wound healing is frequently impaired in patients with diabetes mellitus, and its treatment is still a big challenge. Here, we show that SMF accelerates wound healing in diabetic mice by promoting macrophage polarization to M2 phenotype. Mechanistically, SMF suppressed STAT1-mediated proinflammatory gene expression and facilitated STAT6-mediated anti-inflammatory gene expression in macrophages. Thus, SMF may be a useful therapeutic means for diabetic wound care.

SMF has been applied as a noninvasive and effective therapeutic method in various clinical practices. The low-frequency magnetic therapy has been officially approved by the US Food and Drug Administration (FDA) for orthopedic applications in treating pain and edema in superficial soft tissues, because SMF can modulate cell metabolism, proliferation, and apoptosis [9]. Prolonged period of exposure to SMF may assist the control of hypertension [22]. Meanwhile, SMF exerts a positive role in the treatment of osteoarthritis and nonunion fracture [23–25]. In this study, we found that moderate intensity SMF (0.6 T) markedly improved the wound healing in the genetic mutation-induced type 2 diabetes mouse model. Consistent with our observations, even lower intensity of SMF at 180-230 mT shows beneficial effect on wound healing in streptozotocin-induced diabetes in rats [26, 27]. The potential efficacy of optimal intensity SMF on wound healing in diabetic patients warrants further investigation.

The wound healing process can be divided into three phases: inflammation, proliferation, and remodeling [28]. At the beginning of the inflammation phase, M1-like macrophages, also named proinflammatory macrophages, are recruited and aggregated in a large number to eliminate bacteria, foreign debris, and dead cells [29, 30]. As such, massive M1 macrophages infiltrated the wound area by day 3 after injury. SMF increases macrophage infiltration, which aids wound healing at an acute inflammation stage. In some pathological conditions such as diabetes and infection, wounds may fail to achieve sufficient healing due to chronic inflammatory reaction; consequently, the proliferation and remodeling stage of healing would not occur [31]. After SMF treatment, more anti-inflammatory macrophages, referred to as M2 type, were recruited at day 7 and 14 postinjury, which facilitates resolution of inflammation and wound healing. During the process of the wound healing and scar formation at a late stage, vascularization is necessary to provide sufficient oxygen and nutrition. CD31 expression, a vascularization marker, was enhanced in the wound areas at 7 days after SMF treatment. This finding conforms to the previous report that macrophages play particularly important roles in vascularization [32, 33].

The macrophages can be categorized into proinflammatory, prowound healing, and proresolving ones based on their roles in different stages of the wound healing process [34]. The proinflammatory macrophages produce nitric oxide, ROS, IL-1, IL-6, and TNF-*α* [35], which mediate and facilitate the process of inflammation. The prowound healing macrophages produce elevated levels of growth factors such as PDGF, insulin-like growth factor 1 (IGF-1), vascular endothelial growth factor (VEGF), and transforming growth factor *β*1 (TGF-*β*1) [35, 36], which aid in cellular proliferation, granulation tissue formation, and angiogenesis. The proresolving macrophages suppress inflammation via upregulation of IL-10, arginase 1, and TGF-*β*1 [35, 37]. The expression of inflammatory cytokines, iNOS, MCP-1, IL-6, IL-1*β*, and CCR7, was increased in the injured tissue at an acute inflammation stage by SMF, probably due to increased recruitment of proinflammatory macrophages. Notably, anti-inflammatory cytokines, CD206, Fizz1, IL-10, and Arg-1, were increased in wound tissues by SMF at both day 3 and day 7, due to increased M2 transdifferentiation induced by SMF. Moreover, SMF can enhance the secretion of IL-10 and inhibit the secretion of proinflammatory cytokines, such as IL-6, IL-8, or TNF-*α* [14]. IL-10 has been substantiated to promote a regenerative process of wound healing [38], suggesting that SMF can promote resolution of inflammation in the proliferative phase.

Accumulating studies have reported that the alteration in the JAK/STAT pathway may result in impaired wound healing in a diabetes model and promotes alternative activation of macrophage [39–44]. Activated STAT3 induces the upregulation of iNOS expression, increases NO production in keratinocytes, and promotes angiogenesis in the wound tissue [45]. Macrophage function is always impaired in patients with diabetes, such as STAT-6-mediated M2 polarization. In addition, diabetic conditions, such as high glucose, activate STAT-1 signaling transduction [46]. We found that SMF induced upregulation of STAT6 phosphorylation and downregulation of STAT1 phosphorylation in macrophage, demonstrating that SMF may modulate the JAK/STAT pathways.

In summary, our study showed for the first time the effect of 0.6 T SMF on wound healing in diabetic mice. These results indicate that SMF accelerates diabetic wound healing by promoting macrophage polarization and resolution of inflammation through modulation of the JAK-STAT pathway. Therefore, focusing on SMF in therapeutic interventions might be useful for treating diabetic wound by renormalizing the healing process.

## Figures and Tables

**Table 1:** Primers for RT-PCR analysis in mice.

Genes	Forward primer (5′-3′)	Reverse primer (5′-3′)
IL-12	TGGTTTGCCATCGTTTTGCTG	ACAGGTGAGGTTCACTGTTTCT
IL-10	AGCCTTATCGGAAATGATCCAGT	GGCCTTGTAGACACCTTGGT
IL-6	TAGTCCTTCCTACCCCAATTTCC	TTGGTCCTTAGCCACTCCTTC
*β*-Actin	GGCTGTATTCCCCTCCATCG	CCAGTTGGTAACAATGCCATGT
Fizz1	CCAATCCAGCTAACTATCCCTCC	ACCCAGTAGCAGTCATCCCA
IL-I*β*	GCAACTGTTCCTGAACTCAACT	ATCTTTTGGGGTCCGTCAACT
CCR7	TGTACGAGTCGGTGTGCTTC	GGTAGGTATCCGTCATGGTCTTG
MRC1	CTCTGTTCAGCTATTGGACGC	CGGAATTTCTGGGATTCAGCTTC
iNOS	GTTCTCAGCCCAACAATACAAGA	GTGGACGGGTCGATGTCAC
Arg-1	CTCCAAGCCAAAGTCCTTAGAG	AGGAGCTGTCATTAGGGACATC
YM1	CAGGTCTGGCAATTCTTCTGAA	GTCTTGCTCATGTGTGTAAGTGA
MCP1	TTAAAAACCTGGATCGGAACCA	GCATTAGCTTCAGATTTACGGGT

## Data Availability

The data used to support the findings of this study are available from the corresponding authors upon request.
